# Recent advances in organotypic tissue slice cultures for anticancer drug development

**DOI:** 10.7150/ijbs.78997

**Published:** 2022-09-25

**Authors:** Lin He, Chuxia Deng

**Affiliations:** 1 Cancer Center, Faculty of Health Sciences, University of Macau, Macau SAR, China.; 2 Centre for Precision Medicine Research and Training, Faculty of Health Sciences, University of Macau, Macau SAR, China.; 3 MOE Frontier Science Centre for Precision Oncology, University of Macau, Macau SAR, China.

**Keywords:** organotypic tissue slice culture, anticancer drug discovery, individualized treatment, precision oncology

## Abstract

Organotypic tissue slice culture is established from animal or patient tissues and cultivated in an *in vitro* ecosystem. This technique has made countless contributions to anticancer drug development due to the vast number of advantages, such as the preservation of the cell repertoire and immune components, identification of invasive ability of tumors, toxicity determination of compounds, quick assessment of therapeutic efficacy, and high predictive performance of drug responses. Importantly, it serves as a reliable tool to stratify therapeutic responders from nonresponders and select the optimal standard-of-care treatment regimens for personalized medicine, which is expected to become a potent platform and even the gold standard for anticancer drug screening of individualization in the near future.

## Introduction

The development of novel drugs is time-consuming, laborious, and costly. In the United States, it requires ~13 years and between 1.8-2.6 billion US dollars from commencement to regulatory approval by the Food and Drug Administration (FDA) 1. Despite monumental investments, only 5-14% of compounds ultimately manifest therapeutic efficacy plus manageable treatment-related adverse events and are entitled to the permission of clinical administration 1. The attrition rate of anticancer drug development is even higher; the clinical approval rate of new anticancer drugs is only 3-6% 2, which is attributed to pluralistic reasons, in particular, the usage of suboptimal drug screening and testing platforms. The advent of precision oncology provokes the implementation of individualized medicine into clinical practice. A preluding program of personalized drug screening for cancer patients before treatment by using a reliable drug screening platform to select the optimal treatment regimens theoretically may improve the clinical therapeutic effectiveness.

The most commonly used tool for identifying new antitumor drugs is the two-dimensional (2D) culture of monolayer cells, wherein a standardized high-throughput system is offered to dissect the characteristics of specific cell types 3. Although contemporary high-throughput screening systems allow the simultaneous assessment of tens or hundreds of thousands of compounds, 2D monolayer cell culture hardly serves as the gold standard platform for antitumor drug discovery *in vitro* due to its many inherent drawbacks. First, monolayer cell culture rarely recapitulates tumoral heterogeneity and complexity, not to mention the tumor microenvironment (TME) 4. The TME contains physical, chemical, and biological elements around cancer cells, which are responsible for the cell-cell and cell-extracellular environment interactions that can shape many important biological properties of tumors, e.g., cell differentiation, proliferation, viability, genetic and proteinic expression, suppression and promotion of metastasis, and drug metabolism 5-8. Second, the genetic and proteinic expression of tumor cells may be changed during the 2D monolayer cell culture 9. Furthermore, the monolayer morphology interferes with the authentic drug response of cancer cells because there is devoid of multicellular drug resistance 10. In parallel, the monolayer morphology indicates the unlimited accessibility of oxygen, drugs, and nutrients to cells, which cannot mimic the *in vivo* phenomenon of gradient-degressive diffusion and perfusion-controlled delivery.

The three-dimentional (3D) organoid technique has been developed rapidly for the identification of anticancer drugs and personalized medicine in the past decade 11, 12. Organoids are derived from stem cells or progenitor cells and form miniaturized organ-like structures that recreate the chief aspects of the 3D anatomy and multiple cell repertoire of the physiological counterpart and recapitulate basic tissue-level functions and many important characteristics (e.g., mutation spectrum and gene expression) 12-16. In addition, patient-derived organoids have demonstrated high predictive performance to predict the clinical responses of anticancer drugs in diverse carcinomas 17-20. However, constructing organoids from tumor biopsies may meet some vexing problems, such as a low number of tumor cellularity and an unmet success rate 10, 14. This method cannot recapitulate many key cell types in the TME (e.g., immune cells). Another anticancer drug discovery platform, the patient-derived xenograft (PDX), also suffers from several shortcomings, including overlong generation time, high cost, and uncertainty of successful establishment. The successful establishment rate of PDX is contextual and depends on the type and origin of tumors. Nevertheless, it shall be never overlooked the potency of this model in obtaining FDA approval for many drugs.

In 2009, Ootani et al. 21 first introduced a 3D air-liquid interface (3D-ALI) method for an intestinal epithelial culture where the tissue was minced by simple manual scissoring into small pieces (under 0.3 mm^3^) and then mixed with collagen before cultivation at the air-liquid interface. Because of the success in the recapitulation of a Wnt-dependent stem cell niche, differentiation, and ultrastructure of stomach cell lineages 21, 22, they further employed this technique in the *in vitro* tumor tissue culture and strikingly found that the immune cells, T-cell receptor repertoire, and phenotypic and genotypic profiling were highly preserved 23. However, a raised concern is that these undersized minced tissues may not be sufficient to portray the intratumor heterogeneity as it is unclear how many small pieces would be needed to fully recapitulate the heterogeneity of original tumors. Thus, the consistency of drug responses between the 3D-ALI method and the internal condition is required for validation.

The organotypic tissue slice culture platform is also pragmatic for anticancer drug discovery owing to the integration of the alike *in vivo* microanatomy and the easy manipulation of *in vitro* work 24-26. We recently developed a 3D tumor slice culture (3D-TSC) platform that incorporates a lipofuscin autofluorescence feature for the time-course monitoring of drug responses 27. Compared with other mainstreams, the 3D-TSC platform presents a comparable potential for anticancer drug development (**Table 1**). This review attempts to summarize and contextualize the contributions of organotypic tissue slice culture in anticancer drug discovery and emphasizes the likely future development in this field.

## Organotypic tissue slice cultures

**Figure 1:**
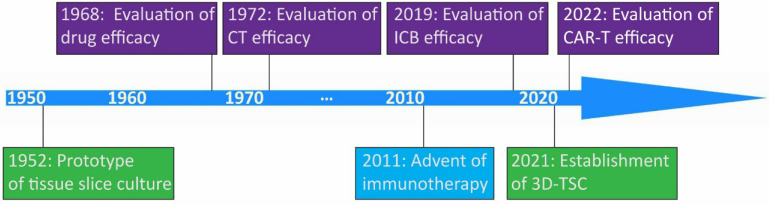
** Milestones in discovery and development of organotypic tissue slice culture.** Key milestones in the prototype of organotypic tissue slice culture and the establishment of three-dimentional tumor slice culture are shown in light green. In 2011, US FDA approved the first immune checkpoint blockade drug, ipilimumab, in the clinical setting. Key milestones in the evaluation of drug efficacy, chemotherapy efficacy, immune checkpoint blockade efficacy, and chimeric antigen receptor T cell therapy efficacy are shown in purple. Abbreviations: CT, chemotherapy; ICB, immune checkpoint blockade; CAR-T, chimeric antigen receptor T cell; 3D-TSC, three-dimentional tumor slice culture.

The prototype of organotypic tissue slice culture was developed by Dr. Harford and coworkers in the 1950s 28. This technique was initially utilized for the assessment of pharmacological efficacy around 1970 29, 30. The expediting evolvement of immunotherapy facilitates the application of this technique to evaluate the immune checkpoint blockade, and adoptive cellular therapy response on tumors in recent years 31, 32 (**Figure 1**). Organotypic tissue slice culture systems represent *in vitro* cultures of explants of patient- or animal-derived tumoral and normal tissues.

### Construction methods

**Figure 2:**
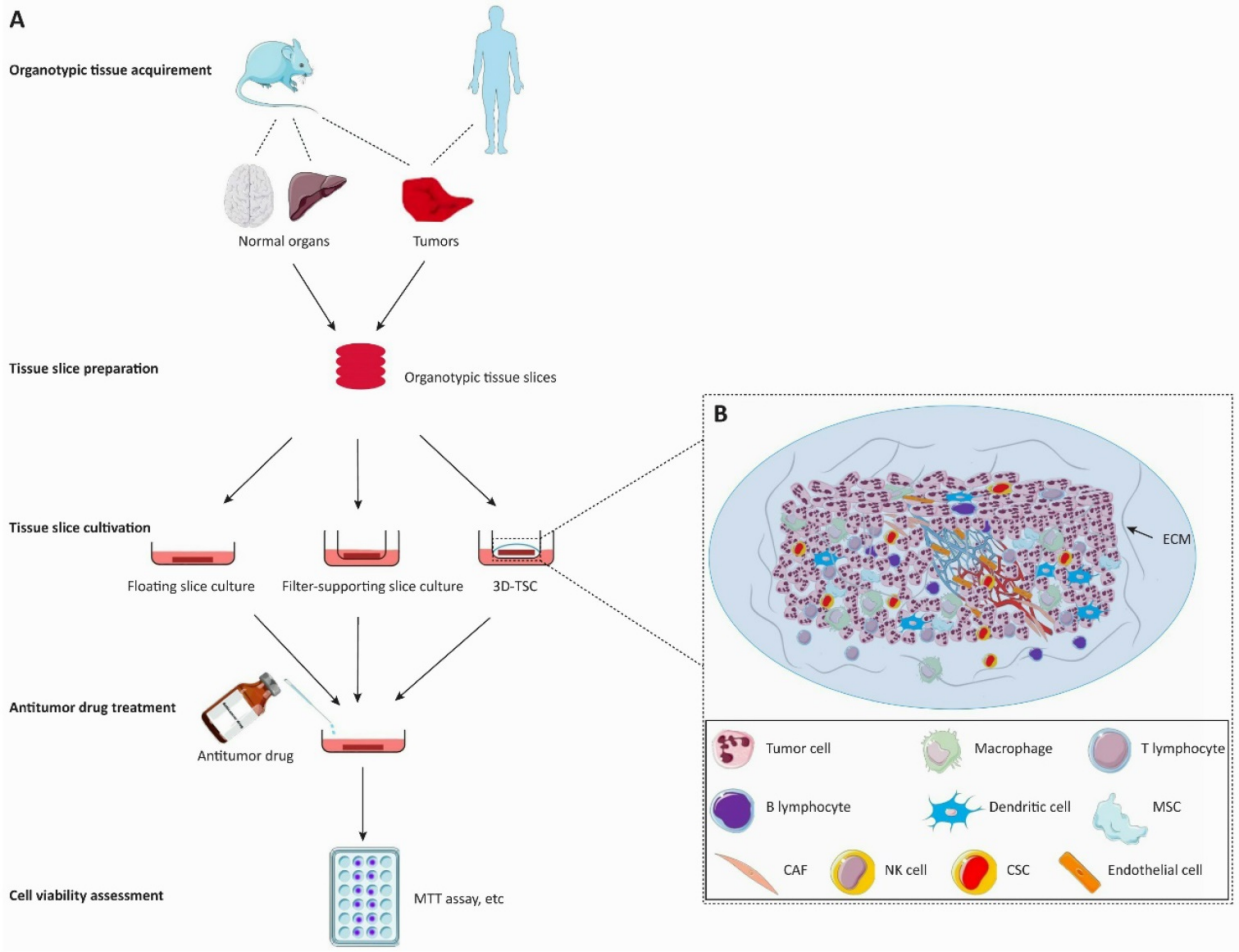
** Flow diagram of the organotypic tissue slice culture system for anticancer drug discovery.** The organotypic tissue slice culture platform can be used to assess the tumoricidal efficacy of anticancer drugs. **A.** In the left panel, animal or patient-derived organotypic tissues are cut into the same figurate slices for the antitumor activity assessment of cancer drugs. Tumor slices are employed to evaluate the tumoricidal efficacy of anticancer drugs whereas normal tissue slices are used to observe the invasive ability of tumors and the efficacy of drugs against tumor invasiveness. Drug treatment is immediately initiated after the generation of the organotypic tissue slice culture system. The cell viability of slices is assessed on the 2-7th day of cultivation. **B.** The 3D-tumor slice culture (3D-TSC) system preserves the architecture and cell repertoire of the original tumor. This platform maximizes the retention of inter-tumor and intratumor heterogeneity, cellular-stromal interactions, and the complexity of the original tumor. The blood vessels on the slices will collapse within a short period after the cessation of blood circulation. Abbreviations: 3D-TSC, three-dimentional tumor slice culture; ECM, extracellular matrix; MSC, Mesenchymal cell; CAF, cancer-associated fibroblast; NK cell, natural killer cell; CSC, cancer stem cell.

The preparation of tissue slice culture mainly involves the following steps (**Figure 2**). Briefly, the surgically resected tissues are collected and placed into cold media, which then are meticulously cut into cylindrical or cuboid shapes. These cylindrical or cuboid tissues are sectioned via a slicing method (e.g., vibratome, precision tissue-slicing machine, or simply manual slicing) under sterile conditions within ~6 hours after tumor excision. Only well-shaped slices are selected for cultivation. Slices were incubated in a humidified incubator at 37 °C and 5% CO_2_ for several days, and the culture media were changed every 2-4 days.

### Underpinning for anticancer drug discovery

Organotypic tissue slice cultures are reliable surrogates for patients or animal models to study some *in vivo* biological characteristics 33-38. One tumor bulk can be sliced into tens or even hundreds of slices, enabling the simultaneous assessment of multiple anticancer drugs and the easy realization of 3Rs (i.e., reduction, replacement and refinement) in experiments. Microinjection of tumor cells into normal tissue slices or implantation of 3D tumor cell spheroids on the surface of those slices (hereon called tumor invasive tissue slices) can recapitulate the tumor migration behaviors, contributing to the discovery of effective antitumor drugs against tumor invasion.

**Figure 3:**
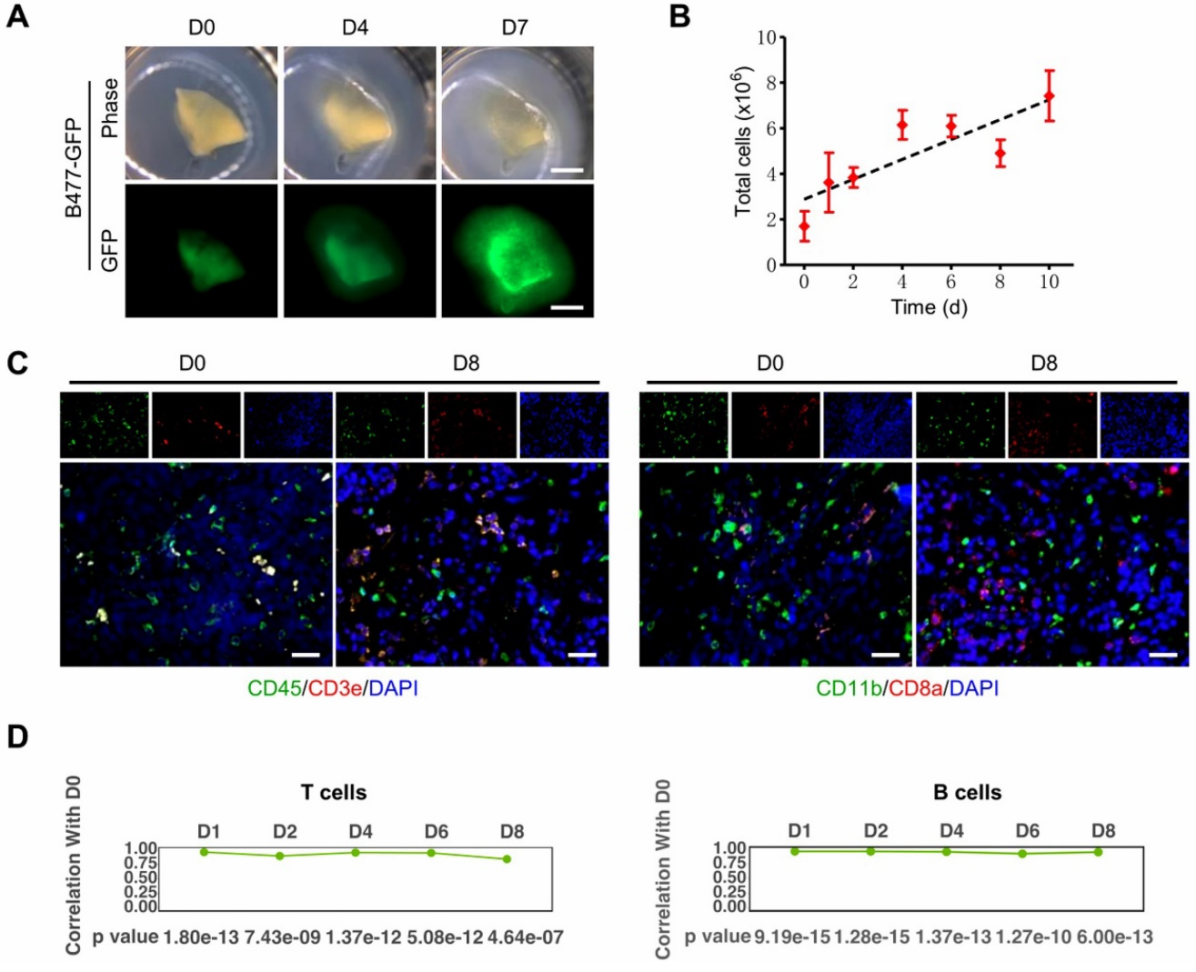
** 3D-tumor slice culture increases the number of live cells and maintains the immune components of their original tumors.** This figure was modified from our previous publication 27 and gained approval from the correspondence authorship. **A.** Observation of tumor growth in 3D-TSCs derived from primary tumors formed by B477-GFP cells injected into the mammary fat pad of nude mice. Scale bar: 1 mm. **B.** Time-lapse of cell viability in 3D-TSCs derived from B477-GFP mouse tumors. **C.** Immunofluorescence staining of immune biomarkers derived from genetically engineered *Brca1Co/Co; MMTV-Cre* mouse in immunocompetent hosts. Blue is nuclear counterstaining by hematoxylin, and brown staining is positive protein staining by DAB. CD3e/CD8a: T lymphocytes; CD11b, macrophages and microglia marker; CD45, T, NK, dendritic, and lymphokine-activated killer cells marker; Scale bar: 100 µm. **D.** Pearson correlation coefficient identifying the correlation of gene expression between Day 1 to Day 8 with Day 0 in T cells and B cells.

The organotypic tissue slice culture platform can be used to validate results obtained from 2D culture and recapitulate *in vivo* circumstances during cell proliferation and metastasis 39, 40. It maximally retains many aspects of the original tumors, including inter-tumor and intratumor heterogeneity, TME, morphology, cellular-stromal interplay, and complexity 24, 41-45. The 3D-TSC platform experiences expanding advantages in these regards 27. First, the slice kept growing for at least 10 days with a gradually increasing number of total live cells (**Figure 3A-B**). Second, the key immune cell repertoire and the gene expression level of T lymphocytes and B lymphocytes can be entirely preserved for at least 8 days (**Figure 3C-D**).

### Optimal parameters of the influencing factors

The generation procedures and culture conditions affect the viability of organotypic tissue slices. Slicing tools, thickness, postresection culture timeframe, and oxygen levels all determine the quality and live cell quantity of slice from the beginning of cultivation to the endpoint of drug assessment. Carefully optimizing the preparation and culture conditions can minimize the artificially induced variations in cell viability.

Most studies generate slices with a thickness of 250-500 μm 25-27, 33, 45-47. Slices with less than 200 μm thickness are extremely fragile and curly and contain large necrotic areas; even at a thickness of 250 μm, a necrosis gradient across the slice has yet been observed 45. No publication has applied tumor slices with a thickness of more than 500 μm for anticancer drug discovery due to the limited provision of oxygen and nutrients to the center area that can lead to tissue necrosis.

Various postresection culture timeframes give rise to slices with diverse persistence to preserve the tissue architecture and viability, ranging from 4 to 16 days 27, 33-36, 46-49. Merz et al. 48 sliced glioblastoma specimens and transferred the slices into the culture system within a few minutes after surgical biopsy and, surprisingly, found that the viability and main histological features of the original tumors were conserved for at least 16 days.

Appropriate oxygen levels are necessary for the retention of slice morphology 26, as hypoxia (< 5% O_2_) elicits rapid and significant changes to many stress pathways and is associated with a negative impact on the morphology of tumor slices 26. Moreover, hyperoxic conditions (41% O_2_) do not increase the tissue viability, metabolic activity, and proliferation of slices compared to normoxic conditions (21% O_2_) 25. Therefore, the atmospheric oxygen level is sufficient for slice culture. Intermittent exposure of slices to oxygen and nutrients using a rotating incubation unit further potentiates slice viability compared to the floating or stagnant filter-supporting slice culture 45, 47. One explanation is that the rotating incubation units result in a higher elimination efficiency of waste products. Floating slices in the liquid culture medium essentially cannot meet the oxygen supplement because of the limited solubility of oxygen (< 2.2 mmol/L) 50. Positioning the slice on a filter at the air-liquid interface achieves the efficient uptake of oxygen and nutrients 27, 33. Koch et al. 50 floated a liver slice on perfluorodecalin, a non-water-soluble chemical of the artificial oxygen carrier, and overlaid it with the culture medium also conquers the predicament of insufficient oxygen supplementation in the culture medium and prolongs the lifespan of the slice as the perfluorodecalin can continuously provide oxygen to the slice from the bottom.

The optimal time window for the assessment of therapeutic efficacy needs to be clarified, which should obviate the slicing-caused activation of stress and inflammatory responses in the beginning and the tissue destruction and devitalization of overlong cultivation. Metabolic activity is continuously elevated within the first 24 hours of cultivation but does not fluctuate at later time points 25. Moreover, slicing-caused tissue damage mainly occurs at the outer part of the slices within the first 24 hours but thereafter is not observed during the subsequent 72 hours of follow-up 25. These findings suggest that the first 24 hours is the contraindicated period for the assessment of therapeutic responses. Also, consistency, high-throughput screening, intratumoral heterogeneity, antibody delivery, cryopreservation, and live tissue imaging are some of the challenges of this system that should be addressed by a balanced review but not the purpose of this work.

## Promising applications

The organotypic tissue slice platform allows the selection of optimal treatment regimens for individualization in the context of precision oncology and has won popularity in the elucidation of tumor invasive ability, the potential toxicity of drugs, and the efficacy assessment of multiple treatment paradigms.

### Identification of tumor invasiveness

The tumor invasive tissue slices visualize the tumor invasiveness in near real-time and offer easy maneuverability for testing the pharmacological efficacy of therapies against tumor migration 51-54. Tumor invasiveness varies with the different morphologies of tumor cells, in which the invasive phenotype cells are characterized by a small soma with a distinct leading process 55. The intratumoral composition of the invasive phenotype cells and the signaling between them and the tumor core are the determinants conditioning tumor invasiveness 55. Therefore, finding a treatment that targets the invasive phenotype cells may significantly mitigate tumor migration.

A profound marker (i.e., “generalized stiffness”) of the invasive phenotype tumor cells has been found on the tumor invasive normal tissue slice culture system 56, 57. Anticancer drug-caused alterations of the tumor cell mechanical and migratory patterns in this model are the theoretical supports for its clinical application in testing the therapeutic efficacy of drugs inhibiting tumor invasion. The mechanical and migratory properties of glioblastoma (GBM) cells on hippocampal slices can be weakened by cannabinoids 57. Similarly, 2 Gy of nonlethal irradiation on this platform attenuates GBM cell invasiveness by increasing the “generalized stiffness” and inducing changes in the actin cytoskeleton and the motility of tumor cells 56. Implanting C6 GBM cells on the surface of brain slices manifests the antitumor invasiveness activity of the Rac1 inhibitor NSC23766)** (Table 2)** 58. The tumor invasive normal tissue slice culture system with these unique advantages will account for more room in the preclinical stage of unveiling many effective antitumor invasive drugs.

### Toxicity determination

Plus, the tumor invasive tissue slices show a promising outlook in the evaluation of the potential pharmacological toxicity. Brain slices can serve as a tool to reflect the toxicity of various neurotoxic compounds and may provide interventions to mitigate toxicity 59, 60. A great predictive performance in the prediction of drug-induced seizure liability is also observed in this model, with 93% sensitivity and 100% specificity 61. The organotypic hippocampal slices have underpinned several mechanistic insights underlying ethanol dependence and/or ethanol withdrawal-induced neurochemical toxicity, such as abnormal synaptic transmission and CA1 pyramidal cell death 62, 63. Drug-induced cholestasis can be modeled on liver slices via 48 hours of incubation with human cholestasis-causing drugs, allowing the observation of the pathogenesis and progression of the disease under an *in vitro* condition 64. Doxorubicin and trastuzumab both show cardiotoxicity in the clinical treatment of breast cancer patients, and this phenomenon is also recapitulated on the heart slice culture model, as manifested by a loss of cardiomyocyte structure and function after 48 hours of treatment 65.

### Assessment of therapeutic efficacy

Organotypic tumor slice culture demonstrates the suitability to investigate the antitumor activity of small-molecule drug therapy (i.e., chemotherapy and molecularly targeted therapy) 24, 25, 27, 41, 47, 49, 66-84, immunotherapy 27, 31, 85, radiotherapy 86-88, and adoptive cellular therapy 32, as summarized in **Table 3** and discussed below.

#### Small-molecule drug therapy

Organotypic slices treated with small-molecule drugs show concentration-dependent and heterogeneous responses 77, 86, exemplified by reduction of cell viability 79, decrease in cell proliferation 67, 80-82, and increase in cell loss and apoptosis 67, 80-82. Tumor slice culture has been incorporated into the discovery of new combination therapy regimens and the efficacy assessment of clinical standard-of-care treatment for multiple cancers 47, 66-68. For example, the oncological efficacy and safety profiles of the CAF regimen (i.e., cyclophosphamide, adriamycin plus 5-fluorouracil) in breast cancers have been confirmed by many landmark phase 2 to 3 randomized controlled trials 89-93, and therefore this regimen is recommended for clinical application by the National Comprehensive Cancer Network (NCCN) Guidelines version 2. 2022 (https://www.nccn.org/professionals/physician_gls/pdf/breast.pdf). Utilizing breast cancer patient-derived slices to assess the CAF response succeeds in the stratification of CAF responders from nonresponders 47. Despite the tumor slices are ubiquitously suitable to assess small-molecule drug responses, it is more intriguing to characterize the immunotherapeutic efficacy.

#### Immunotherapy

Deciphering immune-oncology on organotypic tissue slice was initially described by Sivakumar et al. 31, who demonstrated that the live immune cell populations lasted for 7 days on the filter-supporting tumor slice cultures. Although some immune cell populations are changed between the slice model and the original tumor and the proportion of the immune components are variable with the time-lapse, this technique observed the response of immune checkpoint blockade. Introducing novel biological materials and techniques into the tumor slice culture system may avoid the variation of immune cell populations and key immune mediators, contributing to the more confidential *in vitro* results of immunotherapeutic efficacy. Indeed, Voabil and colleagues 85 embedded patient-derived tumor fragments into an artificial extracellular matrix and found that the lymphocyte efflux was significantly lower than those cultured in medium or collagen, with no significant difference in T-cell functionality and nonspecific immune activation. They evaluated the programmed cell death protein 1 (PD-1) blockade response of 37 tumors from five cancer types (i.e., melanoma, non-small-cell lung cancer, breast cancer, ovarian cancer, and renal cell carcinoma) on this platform. Only a small proportion of tumors (13 out of 37) showed discernable immunotherapy responses, predominantly from melanoma and non-small-cell lung cancer, which was concordant with the clinical outcomes.

The filter-supporting tumor slice culture model with a 0.4 μm pore size membrane culture insert cannot prevent the horizontal lymphocyte efflux and thus may identify the negative result of pembrolizumab (a humanized anti-PD-1 monoclonal antibody) responses 41. Tumor slice in the 3D-TSC platform is precoated with a reconstituted collagen solution and supported by an insert at the air-liquid interface; therefore, the horizontal and vertical lymphocyte efflux is efficiently spared and the lymphocytes are well preserved within 8 days of cultivation 31. We utilized this technique to evaluate the immunotherapeutic efficacy of colon cancer or breast cancer patients. The expression level of programmed cell death ligand 1 (PD-L1) in colon cancer patients was low, only between 2-11%. Patients with 11% PD-L1 expression showed the highest PD-1/PD-L1 blockade response; intriguingly, the anti-PD1/PD-L1 therapeutic efficacy was higher in patients with 2% PD-L1 expression than in those with 5% or 7% PD-L1 expression. One explanation is that intratumoral PD-L1 is heavily glycosylated, and the immunohistochemical readout results may not reflect the reality of PD-L1 expression 94. Removal of N-linked glycosylation significantly elevates PD-L1 detection in human tumor tissues, and the improved PD-L1 detection level is closely related to the clinical oncological efficacy of anti-PD-1/PD-L1 therapy 94. Therefore, PD-L1 after deglycosylation can be used as a biomarker to predict immunotherapeutic efficacy.

#### Radiotherapy

Radiotherapy reduces the risk of tumor relapse, ameliorates survival benefits, and has become an irreplaceable component of systematic treatment for many cancers 95. Organotypic tissue slices exposed to irradiation observe the stall of cell growth and the decrease in tumor volume 86. Cancer stem cell status and DNA damage repair both dictate tumor radioresistance. After 24 hours of exposure to 4 Gy irradiation, the expression levels of the cancer stem cell marker CD44 and DNA damage repair markers γ-H2AX and p-ATM in tumor slices from radiotherapy-sensitive oral squamous cell carcinoma patients are significantly higher than those from radiotherapy-resistant patients 87, suggesting that this platform is applicable to stratify radiotherapeutic responders from nonresponders. Radiotherapy plus the inhibitor of cyclin-dependent kinases 4 and 6 (CDK4/6i) palbociclib contribute to a significantly increased cell death in meningioma slices than either of the monotherapy 88. These results offer preclinical evidence for the cotreatment of CDK4/6i with radiotherapy for meningiomas.

#### Adoptive cellular therapy

Adoptive cellular therapy is a new type of therapy that aims to leverage the internal immune system to obviate cancers, wherein immune cells are engineered with the expression of anti-specific T-cell receptors or chimeric antigen receptors (CARs) that can better recognize and kill malignant cells 96, 97. The overwhelming triumph of CD19-targeted CAR-T cell therapy in refractory B-cell malignant lymphoma is an important landmark of adoptive cellular therapy in cancers 98, 99 and serves as an exemplification for developing more treatment paradigms, e.g., CAR-NK cell therapy 96. The tumor slice culture platform recently has been used to assess the efficacy of CAR-T cell therapy in solid tumors. Despite the low success of CAR-T cell therapy in human colorectal cancer liver metastases (CRLM), the interleukin-10 blockade has shown functions to enhance the antitumor activity of CAR-T cell therapy in human CRLM slices, as characterized by the increased CAR-T cell activation and CAR-T cell-caused cytotoxicity as well as amplified CAR-T cell proliferation 32. Therefore, harnessing tumor slice culture models may broaden the clinical application of adoptive cellular therapy in solid tumors.

## Conclusions and future perspectives

Organotypic tissue slice culture platform has many advantages for anticancer drug discovery, including (1) identification of tumoricidal efficacy of anticancer drugs, specifically the immune checkpoint blockade antibodies; (2) high predictive performance of drug responses; (3) quick stratification of treatment responders and nonresponders; and (4) ascertainment of the cooperativity of different combination therapy strategies. Therefore, this technique may tailor the best clinical guideline-recommended regimens for individualization, providing a decision aid for clinicians. Recapitulation of the complexity and TME of original tumors on the slices is of pivotal importance, particularly the indispensable components for specific anticancer drugs (e.g., immune cell repertoire for PD-1/PD-L1 blockade). Thus, it needs to consider all aspects of the generation and cultivation conditions for maximally maintaining the consistency of the slices with the original tissues. Notably, accurately judging therapeutic efficacy requires averaging an adequate number of slice results due to intratumoral heterogeneity. In addition, the system is not a reproducible tool similar to 3D-ALI or patient-derived xenografts and is normally unable to test a plethora of drug responses. It is crucial to enhance the efficiency of clinical translation of preclinical findings on this technique, for which it needs to narrow down the drug candidates by prescreening compounds by the 2D monolayer cell culture or selecting agents according to the NCCN clinical practice guidelines in oncology.

Different tissue origins and preparation procedures bestow the platform with the intrinsic advantages and limitations in the field of anticancer drug discovery. We enumerated the overlapping but distinct preparation conditions for different organotypic tumor slice models in **Table 4**. Each of them has been documented with the potential to assess the oncological efficacy of the antitumor treatment strategy. In addition, tissue slices have implicated the availability of uncovering novel biomaterials within the synthesis of anticancer drugs; for example, graphene quantum dots and nanoparticles both show complete penetration and minimal viability damage on slices 100, 101, indicating their promising application for the construction of drug delivery systems.

## Figures and Tables

**Table 1:** Overview of the tumor models for anticancer drug discovery

Models	Cell culture	Organoid	PDX*	3D-ALI	3D-TSC
Successful establishment rate	Low	Moderate	Variable	High	High
Generation time	Moderate	Moderate	Long	Short	Short
The minimal tumor size requirement	Small	Small	Big	Small	Big
TME recapitulation	-	-	-/++	++	++
Multicellular drug resistance	-	++	++	+	++
Intact morphology	-	-	++	+	++
Reproducibility	++	++	++	-	-
High-throughput drug screening	++	+	-	-	-

The inapplicability is marked with “-”, the applicability is marked with “+”, and the robust applicability is marked with “++”. *PDX can recapitulate tumor microenvironment from the humanized mice rather than the severe immunodeficient mice. Abbreviations: PDX, patient-derived xenograft; 3D-ALI, three-dimentional air-liquid interface method; 3D-TSC, three-dimentional tumor slice culture; TIME, tumor immune microenvironment.

**Table 2:** Contributions of organotypic tissue slice model from normal organs in antitumor invasive drug discovery

Models	Sketch diagram	Contributions	Reference
Organotypic brain slice culture	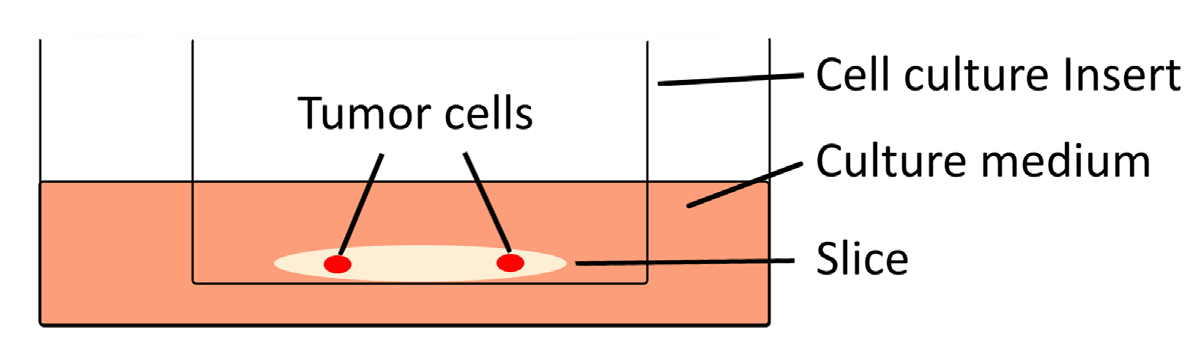	Jasplakinolide, Rac1 inhibitor NSC23766, and tranilast significantly decrease the tumor cell invasion on brain slices.	52, 58, 77
Organotypic cerebellar slice culture	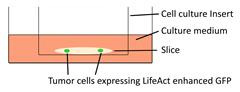	Epidermal growth factor accelerates the invasion of medulloblastoma cells on cerebellar slices.	53
Tissue-based liver-kidney-on-a-chip	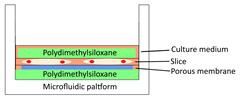	A CXCR4 small-molecule antagonist AMD3100 effectively halts the liver tropism of breast cancer extracellular vesicles.	54
Organotypic hippocampal slice culture	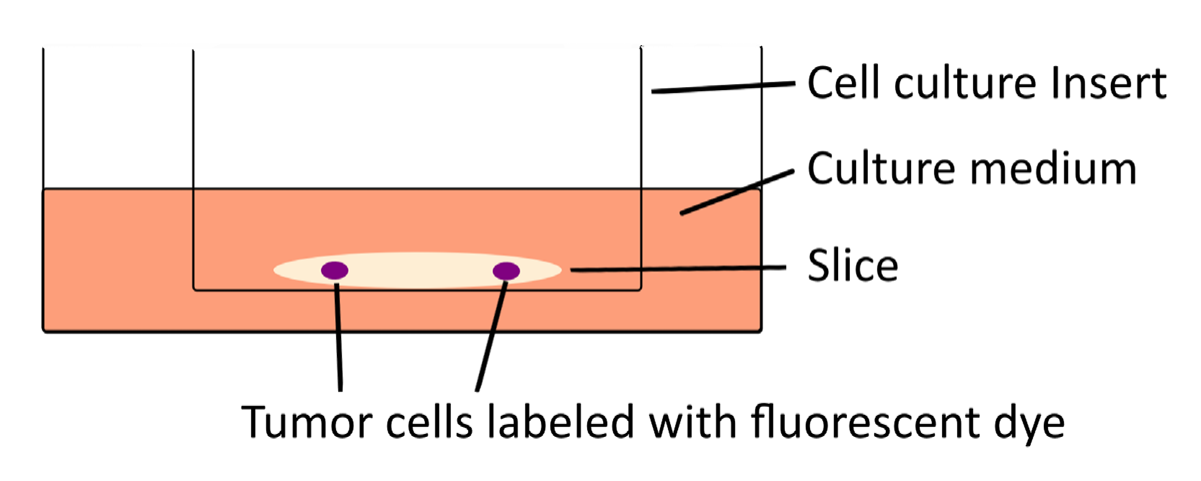	Cannabinoids influence the migratory and mechanical properties of tumor cells on organotypic hippocampal slices.	57

**Table 3:** The *in vitro* response of treatments on the patient-derived tumor slice culture system

Type of cancer	Treatment*	No. of patients	*In vitro* response	Reference
Prostate cancer and bladder cancer	Docetaxel or gemcitabine	10	Induction of cell death and increase in cell loss	24
Pancreatic ductal adenocarcinoma	Rapamycin	12	Decrease in metabolic activity	25
Colon cancer and breast cancer	Chemotherapy, endocrinotherapy, targeted therapy, immunotherapy, and polytherapy†	7‡	Decrease in cell viability and increase in apoptosis, with a heterogenous individual response to chemotherapy or immunotherapy.	27
Colorectal cancer liver metastasis	IL-10 antibody plus CAR-T cell therapy	38	αIL-10 augments CAR-T cell activation and CAR-T cell-mediated cytotoxicity	32
Hepatic metastatic colorectal carcinoma	Oxaliplatin, cetuximab, or pembrolizumab	9	Decrease in cell proliferation, with a heterogenous individual response to chemotherapy and targeted therapy	41
Breast cancer	Cyclophosphamide, adriamycin plus 5-FU	15	Decrease in cell proliferation and induction of cell death	47
Glioblastoma	Temozolomide	12	Decrease in cell proliferation and increase in cell loss and apoptosis, with a heterogenous individual response to chemotherapy	48
Gastric and esophagogastric junction cancer	5-FU or cisplatin	13	Increase in cell loss and apoptosis	49
Hepatocellular carcinoma	Sorafenib plus N20 blocking peptide	13	Decrease in cell proliferation	66
Colorectal carcinoma	5-FU	7	A dose-dependent decrease in cell proliferation, with a heterogenous individual response to chemotherapy	67
Bladder cancer	Mitomycin-C plus coxsackie A21	1	Stronger apoptosis in the combination therapy than either of the monotherapy	68
HNSCC	Cetuximab	10	Decrease in cell proliferation, with a heterogenous individual response to targeted therapy	69
HNSCC	Cetuximab	14	Decrease in cell proliferation, with a heterogenous individual response to targeted therapy	70
Glioblastoma	Gefitinib	1	Insensitive anticancer activity	71
Melanoma	Ribociclib plus CGM097	13	The impedance of cell growth	72
Prostate cancer	Enzalutamide, or olaparib	3	Decrease in cell proliferation and increase in cell loss, with a heterogenous individual response to anti-androgen or targeted therapy	73
Breast cancer	Rapamycin	30	Decrease in cell proliferation, with a heterogenous individual response to targeted therapy	75
Rectal cancer liver metastasis	Oxaliplatin	20	Decrease in tumor size and cell viability, and increase in apoptosis	78
Breast cancer	Doxorubicin	1	A dose-dependent decrease in cell viability	79
Pancreatic ductal adenocarcinoma	Staurosporine, gemcitabine or cisplatin	10	Decrease in cell proliferation and increase in cell loss and apoptosis	80
Pancreatic ductal adenocarcinoma	Staurosporine or cycloheximide	13	A dose- and time-dependent increase in apoptosis and decrease in cell proliferation	81, 82
Lung cancer	Cisplatin	32	Induction of cell death	83
Melanoma, NSCLC, RCC, breast cancer, and ovarian cancer	Nivolumab	37‡	Increase in immune activity, with a heterogenous individual response to immunotherapy	85
Oral squamous cell carcinoma	4 Gy irradiation	28	More cancer stem cells and DNA damage response in responders than nonresponders	87

*The “or”-connected drugs represent monotherapy, while the “plus”-connected drugs represent combination therapy. ‡These 7 patients consist of 2 breast cancer patients and 5 colon cancer patients, and these 37 patients consist of 13 melanoma patients, 7 NSLCC patients, 8 breast cancer patients, 6 ovarian patients, and 3 RCC patients. †The drugs involved in these treatments include 5-fluorouracil, cisplatin, docetaxel, doxorubicin, epirubicin, mitoxantrone, irinotecan, daunorubicin, tamoxifen, neratinib, ceritinib, afatinib, regorafenib, osimertinib, palbociclib, pembrolizumab, durvalumab, and durvalumab plus IL-2. Abbreviations: 5-FU, 5-fluorouracil; HNSCC, head and neck squamous cell carcinoma; RCC, renal cell carcinoma.

**Table 4:** The promising applications of different organotypic tumor slice cultures in antitumor drug discovery

Models	Sketch diagram	Small-molecule drug therapy	Immunotherapy	Radiotherapy	Adoptive cellular therapy	Reference
Filter-supporting tumor slice culture	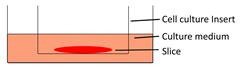	√	√	√	√	25, 35, 41, 49, 66, 81
Floating tumor slice culture	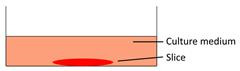	√	√	√	×	26, 33, 68, 69, 72, 75
3D-tumor slice culture	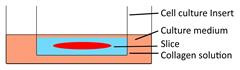	√	√	×	×	27
Tumor slice culture on a rotating platform	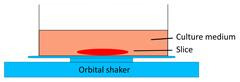	√	×	×	×	47
Collagen-supporting tumor slice culture	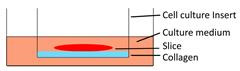	√	×	×	×	80
Tumor slice culture on a microfluidic platform	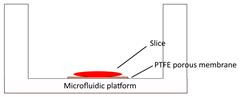	√	×	×	×	84
Patient-derived tumor fragment culture	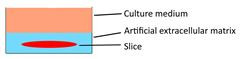	×	√	×	×	85

The application that has been described in the publications is marked with “√”, otherwise is marked with “×”.

## Abbreviations

2D: two-dimensional
TME: tumor microenvironment
3D: three-dimentional
PDX: patient-derived xenograft
3D-ALI: 3D air-liquid interface
3D-TSC: 3D tumor slice culture
GBM: glioblastoma
CAF: cyclophosphamide, adriamycin plus 5-fluorouracil
NCCN: National Comprehensive Cancer Network
PD-1: programmed cell death protein 1
PD-L1: programmed cell death ligand 1
CDK4/6i: inhibitor of cyclin-dependent kinases 4 and 6
CARs: chimeric antigen receptors
CRLM: colorectal cancer liver metastases
